# DNA methylation remodeling in temozolomide resistant recurrent glioblastoma: comparing epigenetic dynamics in vitro and in vivo

**DOI:** 10.1186/s12967-025-06767-x

**Published:** 2025-07-10

**Authors:** Michela Buonaiuto, Mariella Cuomo, Davide Costabile, Federica Trio, Sara Ferraro, Ornella Affinito, Alberto De Bellis, Maria Laura Del Basso De Caro, Roberta Visconti, Lorenzo Chiariotti, Giuseppe Catapano, Rosa Della Monica

**Affiliations:** 1 CEINGE-Advanced Biotechnologies “Franco Salvatore”, Naples, Italy; 2 Department of Molecular Medicine and Medical Biotechnologies, University of Napoli “Federico II”, Naples, Italy; 3 AREA Science Park, Padriciano, Trieste, Italy; 4 Neurosurgery Unit, AORN San Sebastiano and S’Anna, Caserta, Italy; 5 “Maria Rosaria Maglione Foundation Onlus”, Naples, Italy; 6 Pathology Unit, University of Napoli “Federico II”, Naples, Italy; 7 Institute of the Endotypes in Oncology, Metabolism and Immunology “G. Salvatore”, National Council of Research of Italy, Naples, Italy; 8 Neurosurgery Unit, “Ospedale del Mare” Hospital, Naples, Italy

## Abstract

**Background:**

Glioblastoma is the most aggressive type of brain tumor and is associated with a poor prognosis. First-line treatment is surgical resection followed by radiotherapy and temozolomide-based chemotherapy. However, the duration of treatment with temozolomide is limited due to both its toxicity and the development of drug resistance. The prognostic and predictive factor for response to temozolomide is the methylation status of the MGMT promoter. Indeed, loss of MGMT promoter methylation is a major cause of chemoresistance. However, the development of drug resistance is not only associated with changes in MGMT methylation. The entire epigenome changes and acquires specific properties necessary for tumor progression.

**Methods:**

To study epigenetic alterations associated with temozolomide exposure, we generated a TMZ-resistant cell model. We investigated epigenetic alterations in the cell model and in a cohort of patients with recurrent glioblastoma using genome-wide methylome approaches (Epic Arrays 850 k).

**Results:**

We investigated the epigenetic changes associated with temozolomide exposure. Therefore, we generated a TMZ-resistant cell model and studied the epigenetic features acquired after selective temozolomide pressure. Our next step was to investigate the epigenomic differences between primary and recurrent tumors in a small cohort of patients. Finally, we performed a cross-analysis between the epigenetic differences found in TMZ-resistant cells and recurrent glioblastomas to identify common signatures that could be used to guide future resistance-overcoming studies.

**Conclusions:**

Temozolomide induces significant epigenetic changes in glioblastoma, which may contribute to treatment resistance and increased tumor aggressiveness. The results suggest that further research into DNA methylation changes associated with TMZ resistance is crucial. The use of primary tumor cells in resistance models may help identify strategies to overcome chemoresistance in glioblastoma.

**Supplementary Information:**

The online version contains supplementary material available at 10.1186/s12967-025-06767-x.

## Introduction

Glioblastoma (GB) is the most aggressive brain tumor associated with poor prognosis [1–5]. The overall survival after first diagnosis is about 15 months [2] and several factors are associated with the average survival. First, the localization of the primary tumor and thus the possibility of tumor eradication impact on patient survival [5]. Second, the outcome of the disease may depend on the histopathological classification and molecular characteristics of the tumor, in particular O^6^-Methylguanine-DNA Methyltransferase (MGMT) methylation status [3–7]. The MGMT gene encodes for an enzyme involved in DNA damage repair and MGMT promoter methylation is considered a prognostic biomarker since it predicts response to chemotherapy based on temozolomide (TMZ) treatment [3–6]. TMZ is an alkylating agent that upon hydrolysis generates a highly reactive methyl diazonium cation capable of methylating various residues on adenosine and guanine bases leading to DNA lesions and inducing cell death. [5–7] Thus, the MGMT enzyme antagonizes the effects of TMZ by removing methyl residues from the O6 position of guanine. When MGMT promoter is hypermethylated, MGMT protein expression decreases and the chemotherapy works properly. On the contrary, hypomethylation of the MGMT promoter gene is associated with increased MGMT activity and correlates with resistance to TMZ [8, 9]. The use of TMZ is limited both because of severe toxicity and, more importantly, because of the development of drug resistance also in patients with methylated MGMT gene [8, 9]. Several efforts have been conducted to overcome GB resistance to TMZ treatment [10, 11], and several GB cell models have been developed to study TMZ resistance [8, 9, 12]. The most widely used method to mimic chemoresistance to TMZ in vitro is the chronic exposure of GB cell lines to increasing doses of TMZ [9, 10]. Chronic treatment with TMZ induces progressive cellular adaptation and acquisition of genetic and epigenetic alterations, including changes in the methylation status of MGMT. Indeed, a decrease in MGMT methylation is often observed in cell lines chronically treated with TMZ as well as in recurrent glioblastomas. In different GB subtypes, the entire epigenome evolves, so that the genome-wide DNA methylation profile analyzed by the Infinium EPIC array (Illumina) [13, 14] has recently emerged as a powerful tool for precise molecular classification and diagnosis of central nervous system (CNS) tumors [13, 14]. In this context, the genetic and epigenetic alterations acquired in recurrent GB have been studied, and several genes have been found to be differentially methylated in tumor recurrences compared to primary tumors, with a global tendency towards hypermethylation [15–17]. However, the correlation between the epigenomic panorama of recurrent GB treated with TMZ and the in vitro induced TMZ-resistant cell lines is still poorly understood. Therefore, the aim of the present study was to investigate in depth the methylation profile of TMZ-resistant clones obtained from a GB cell line in order to assess common epigenetic signatures within tumor heterogeneity. Then, we evaluated longitudinal changes in the methylation profile of recurrent GBs compared to the primitive tumor. Finally, we performed a cross-analysis between the “episignatures” found in TMZ-resistant clones and the differentially methylated regions found in the recurrent GBs.

## Materials and methods

### Generation of TMZ-resistant glioblastoma clones

LN18 glioblastoma cell line was purchased from ATCC and grown in DMEM (Sigma-Aldrich, St. Louis, MO, USA), as previously described [18]. To generate TMZ-resistant clones, the glioblastoma cell line LN18 was treated with 100 μM TMZ (Selleckchem) or 0.1% DMSO solvent control, changing the medium and adding TMZ every two days to a total of three weeks. TMZ treatment schedule was based on literature evidence [19, 20] and cell survival. LN18 cells were cultured in p100 mm plates. Upon reaching confluence, the plate was collected and diluted by seeding 1/100 of the cell suspension into a p150 mm plate.

We allowed the cells to duplicate until most of the single cells in the plate reached the formation of subclone colonies. Each colony was then picked and seeded into 24-well plates. As the cells reached confluence, they were progressively transferred to larger plates for further expansion. We finally obtained stable cell lines, called clones (C), resistant to TMZ (C1-C2-C3-C4-C5). Concomitantly, we generated LN18 control plates (CT) treated with 0.1% DMSO.

### Patient recruitment

Primary (n = 4) and recurrent (n = 4) tumor samples were obtained from “Ospedale del Mare” in Naples. The study was approved by the Ethical Committee of the University of Naples “Federico II” (number 56/21). Written informed consent was obtained from the patients. Characteristics of tumors are shown in Table 1.

**Table 1:** Characterization of Glioblastoma samples

	Date of primitive resection	MGMT	IDH 1 /IDH 2	Diagnosis	Date of recurrent resection	MGMT	IDH 1 /IDH 2	Diagnosis
GB 1	31/08/2020	Methylated	WT	Glioblastoma IDH WT	22/09/2021	Methylated	WT	Glioblastoma IDH WT
GB 2	16/06/2020	Methylated	WT	Glioblastoma IDH WT	21/11/2022	Unmethylated	WT	Glioblastoma IDH WT
GB 3	04/12/2020	Methylated	WT	Glioblastoma IDH WT	08/10/2021	Unmethylated	WT	Glioblastoma IDH WT
GB 4	30/12/2022	Unmethylated	WT	Glioblastoma IDH WT	24/06/2023	Unmethylated	WT	Glioblastoma IDH WT

### mRNA expression analyses

RNA of each sample was extracted using RNeasy Mini Kit (QIAGEN, Hilden, Germany) following the manufacturer’s instructions. Then, 2 μg of the extracted RNA was reverse-transcribed using QuantiTect Reverse Transcription kit (QIAGEN) following the manufacturer’s instructions. qPCR were performed using LightCycler 480 SYBR Green I Master (Roche Diagnostic, F. Hoffmann). The reactions were performed in a LightCycler480 RealTime thermocycler. The following protocol was adopted: 10 s for initial denaturation at 95 °C followed by 40 cycles consisting of 30 s at 94 °C for denaturation, 30 s at 60 °C for annealing, and 30 s for elongation at 72 °C (final elongation). The following primers were used to assess MGMT transcription levels: MGMT FW 5ʹ-GCTGAATGCCTATTTCCACCA-3ʹ MGMT; RV: 5ʹ-CACAACCTTCAGCAGCTTCCA-3ʹ; GAPDH was used as housekeeping gene for qPCR using the following primers: GAPDH FW: 5ʹ-AGCCACATCGCTCAGACAC-3ʹ; GAPDH RV: 5-GCCCAATACGACCAAATCC-3. qPCRs were performed in triplicate and repeated three times. As reported in supplementary Fig. 2, we also evaluated MEF2B and MAPK6 mRNA expression levels by qPCR in Clones vs CTs. For this purpose, we used the following primers: MEF2B FW: 5ʹ -ATGGACCGTGTGCTGCTGAAGT-3ʹ; MEF2B RV: 5ʹ-TCCGAAACTTCTCTCCTGGCTC-3ʹ; MAPK6 FW: 5ʹ - GACATGACTGAGCCACACAAACC-3ʹ; MAPK6 RV: 5ʹ -GATGGGAGAGTGCTTCTTCTGC-3’.

Furthermore, we obtained RNA-seq data from patients with primary and recurrent glioblastoma Gene expression data from 25 patients with primary and 18 recurrent glioblastoma were obtained from Gene Expression Omnibus (GEO) data repository (GSE62153). Raw data were processed and normalized using the limma package in R. Expression values for the MGMT gene were extracted and compared between primary and recurrent glioblastoma samples. All samples were derived from different individuals (i.e., no matched pairs). Data were visualized using box plots, and group differences were assessed using the Wilcoxon rank-sum test.

### Western blot analyses

Cells were collected and lysed. Lysis buffer was prepared using 0.1% NP40-PBS, plus phosSTOP and Complete Protease inhibitor (Roche Life Science, Penzberg, Germany). Lysates were put on ice for 10 min and centrifuged at 14,000 × *g* for 20 min. Protein lysates were separated on SDS/PAGE and blotted. Western blots were repeated three times. We purchased the following antibodies: anti-α-tubulin (Abcam, Cambridge), anti-MGMT (Santa Cruz Biotechnology, Texas), anti-MEF2B (Santa Cruz Biotechnology), anti-MAPK6 (Santa Cruz Biotechnology). Secondary antibodies were purchased from Thermo Fisher Scientific: Goat anti-Rabbit IgG (H + L), HRP, and Goat anti-Mouse IgG (H + L) Secondary antibody, HRP. Filters were finally read using ChemiDoc™ Imaging System (Biorad, California).

### DNA extraction and bisulfite conversion

DNA from CT and Clones was extracted using the DNeasy blood and Tissue Kit (QIAGEN) according to the manufacturer’s instructions. DNA from primary and recurrent tumors was extracted using the FFPE DNA Tissue Kit (QIAGEN) following the manufacturer’s instructions. Total DNA amount was measured using NanoDrop*One* (Thermo Scientific, Waltham, MA, USA). Genomic DNA (500 ng) was converted by sodium bisulfite using the EZ DNA Methylation Gold Kit (Zymo Research, Irvine, CA, USA) and eluted in 50 μl of RNase-free water according to the manufacturer’s instructions.

### Methylation-specific PCR (MSP) analysis

The methylation of the MGMT gene promoter was assessed by Methylation Specific PCR (MSP), as previously described [21]. We performed a nested PCR using bisulfite-specific primers and a bisulfite-treated DNA template. The first PCR was performed using the following primers: forward (PAN forward: 5′-GGATATGTTGGGATATAGTT-3) and reverse (PAN reverse: 5′-CCATCCACAATCACTACAAC-3). After the first PCR, the amplicons were subjected to a second PCR, that was performed using specific primers to distinguish between methylated and unmethylated DNA. To recognize MGMT methylation, we used the following primers: forward, 5′-TTTCGACGTTCGTAGGTTTTCGC-3′; reverse, 5′-GCACTCTTCCGAAAACGAAACG-3′. To recognize MGMT absence of methylation, we used the following primers: forward, 5′-TTTGTGTTTTGATGTTTGTAGGTTTTTGT-3′;

reverse, 5′-AACTCCACACTCTTCCAAAAACAAAACA3′. We obtained amplicons of 81 base pairs (bp) with specific primers for methylated DNA, and 91 bp with specific primers for unmethylated DNA. MSP allows to measure the qualitative methylation status of MGMT by investigating 9 CpG sites associated to transcriptional regulation of the gene.

We used a fully methylated (CT +) and unmethylated (CT-) DNA as PCR positive and negative controls, respectively. A PCR sample mix, without DNA, was used as a reaction control. MSP products were loaded directly onto a 3% agarose gels, stained with ethidium bromide (Sigma-Aldrich, St. Louis, MO, USA), and viewed under ultraviolet illumination (Bio-Rad, Hercules, CA, USA). We used an appropriate 50 bp DNA ladder (New England Biolabs).

### DNA methylome analyses and data acquisition

Methylome analyses were performed on bisulfite-treated DNA from cells and tumors using the Infinium Methylation EPIC Array 850 K (Illumina, San Diego, CA, USA). We preprocessed the array IDAT intensity data in R statistical environment using the RnBeads pipeline analysis package. Data were filtered by removing probes containing missing values or SNPs. Therefore, the filtered beta values were subjected to differential methylation analyses by using RnBeads package, to obtain differentially methylated genes. Differentially methylated genes were considered significant if the p-value was < 0.05. [22].

### Gene ontology (GO)

Gene ontology (GO) was performed using David tools from National Institutes of Health (NIH). We evaluated the GO of differentially methylated genes obtained from Clones vs CTs and from Recurrent vs Primary tumors analyses, and we represented the top ten most enriched and significant pathways.

## Results

### Generation and epigenetic profiling of TMZ-GB resistant clones

**Fig. 1:**
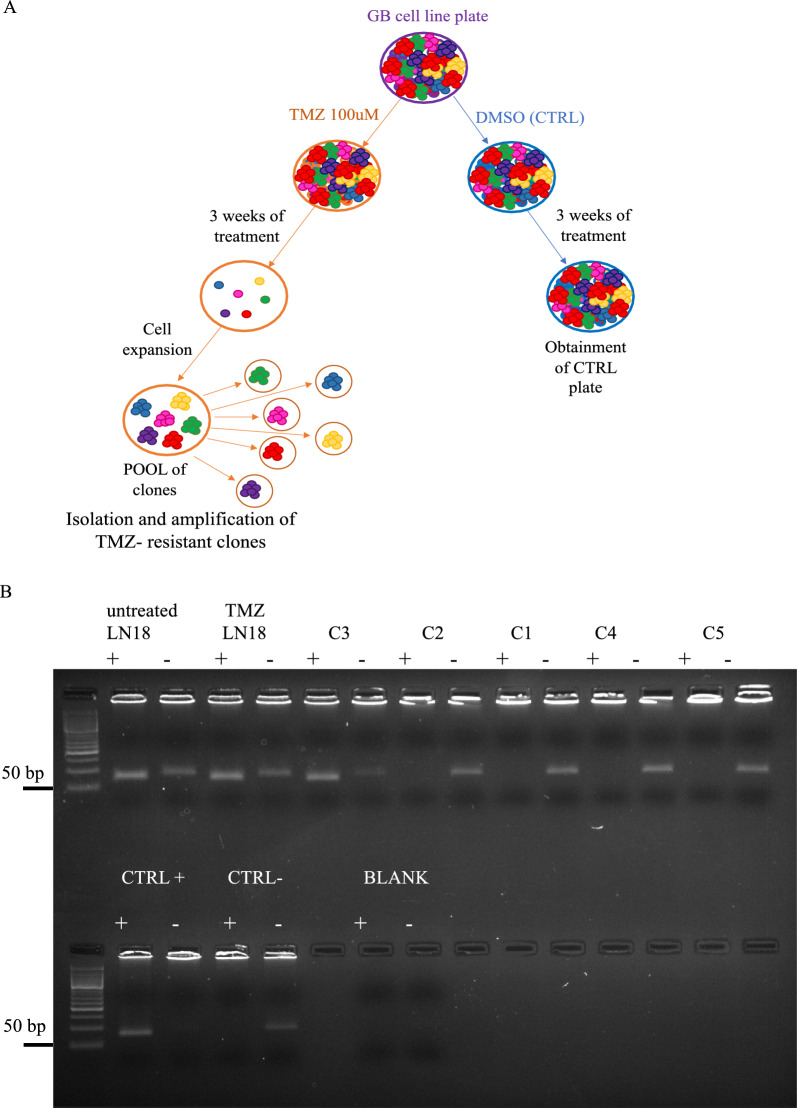
Generation and MGMT detection of TMZ-resistant clones: Fig. 1: **A** Schematic protocol illustrating the generation of TMZ-resistant clones. **B** Evaluation of MGMT methylation by MSP. Electrophoresis on agarose gel of the MSP: untreated LN18 cells (DMSO), LN18 TMZ (LN18 pool chronically treated with TMZ), 2 CT fully methylated, 2 CT fully unmethylated, the Blank control (without DNA), and 5 TMZ-resistant clones. For each sample, the lane signed with + indicates the amplification with methylated DNA-specific primers, the lane signed with—indicates the amplification with unmethylated DNA-specific primers. We used a 50 bp ladder as reported

TMZ-resistant glioblastoma clones were generated by TMZ treatment of the MGMT-methylated GB cell line LN18 as described in the Materials and Methods section and shown in Fig. 1A. For each clone, we evaluated the MGMT methylation status in five TMZ-resistant clones using Methylation Specific PCR (MSP). We found heterogenous status of MGMT methylation and we observed a decrease of methylation in the clones C1-C2-C4-C5, while C3 clone remained methylated (Fig. 1B).

To study all the epigenetic alterations in TMZ clones, in addition to MGMT methylation status, we performed genome-wide methylation profiling using the Infinium methylation EPIC array, including 850,000 probes for CpG methylation detection, in order to identify DNA methylation signatures associated with TMZ resistance.

**Fig. 2:**
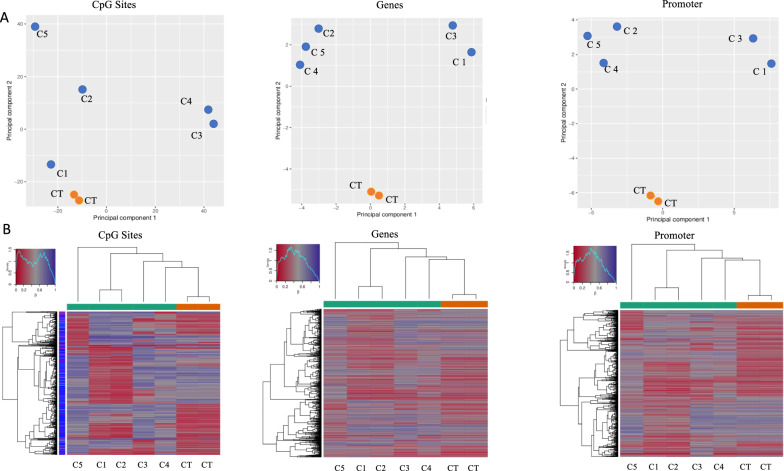
Exploratory analysis of clones vs Cts methylation profiles **A**) Principal Component Analysis (PCA) plots representing the group of TMZ-resistant clones (C) and the group of controls (CT). PCA plots show the cluster of CT group in orange and the disposition of clones in green, respectively. The plots have been generated evaluating the overall differences in DNA methylation levels considering CpG sites, genes and promoters. **B**) Hierarchical Cluster for the group of TMZ-resistant clones (C) and CT group. Heatmaps showing the methylation profiles and methylation differences at selected CpG sites, regions, and promoters with the highest variance across all

We processed IDAT files from the methylome array using R-based RnBeads scripts [23]. To evaluate epigenomic differences between TMZ resistant clones and CT we performed an exploratory analysis by principal component plots (PCA). These included clustering samples based on methylation levels at all analyzed individual CpG sites, and then selecting those located at gene bodies and promoter’s regions, respectively (Fig. 2A). TMZ-resistant clones clustered far away from controls, revealing differences in epigenomic profile.

Then, we performed hierarchical clustering based on the methylation levels at CpG sites, promoters and genes regions exhibiting the highest variance between TMZ resistant clones and CT (Fig. 2 B). Specifically, we observed statistically significant methylation differences between CT and TMZ-resistant clones at the CpG sites, genes and promoter levels.

To assess DNA methylation differences at higher resolution we focused on significantly differentially methylated individual CpG sites (p-value = < 0,05). In particular, we found 66,747 differentially methylated CpG sites, 47,074 CpGs hypermethylated in TMZ resistant clones and 19,674 CpGs sites hypomethylated and, by selecting CpG sites located within genes, we detected 3989 differential methylated genes, most of them hypermethylated in resistant clones (3,268 genes) compared to CT.

**Fig. 3:**
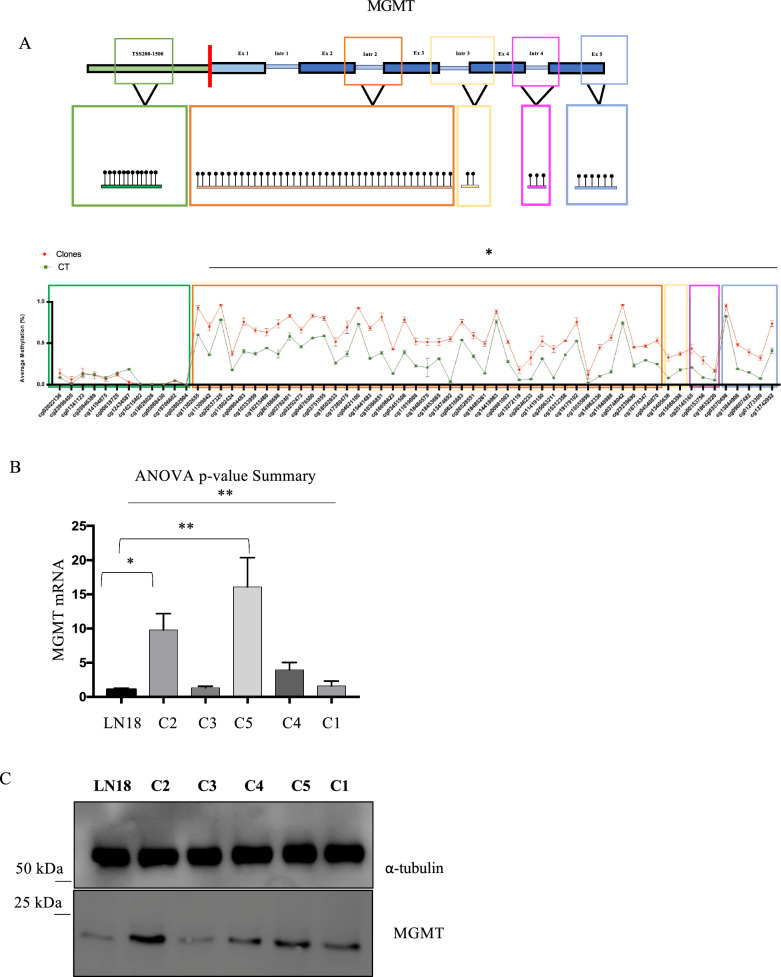
CpG methylation differences in MGMT gene body and TSS upstream region: Clones vs Cts **A**) Schematic representation of the MGMT gene. Upstream regions from transcription start sites (TSS), specifically 200 bp and 1500 bp, are represented in green (TSS200-1500). Exons are represented in blue (ex), and introns are represented in light blue (intr). The TSS is shown as a red bar. Exon 1 is shown in light blue due to its regulatory role in MGMT transcription. The green, orange, yellow, pink, and blue rectangles represent different regions showing changes in DNA methylation within the gene. The CpG coordinates are derived from the hg38 genome. The average methylation per CpG site, with its standard error, is reported. The values detected in Cts are represented in green and the values reported in Clones are represented in red. Statistically significant, differentially methylated CpGs are indicated (*p<0.05). **B**) mRNA expression levels of MGMT were assessed by qPCR. Statistical analyses were performed using one-way ANOVA. All values are given as the mean ± standard deviation of at least three replicates (** p-value summary = 0.0011). **C**) LN18 cells (treated with DMSO) and TMZ-resistant LN18 clones were collected and lysed. The extracted proteins were separated by SDS-PAGE, blotted, and probed for MGMT. Protein levels were normalized by probing for α-tubulin. The molecular weight indicated by the protein ladder was reported

We firstly examined the methylation status of MGMT. Strikingly, we detected 51 significant hypermethylated CpG sites at the MGMT gene in TMZ-resistant clones. In this regard, we mapped the differentially methylated CpG sites and found that all of them were located in the gene body, especially in intron 2 (Fig. 3A). It has been observed that methylation in the gene body is often associated with high gene expression, in contrast to promoter hypermethylation, which is associated with decreased gene expression [24]. To validate this hypothesis, we performed real-time PCR for MGMT and compared MGMT gene expression in CT versus TMZ-resistant clones (Fig. 3B). We found increased levels of MGMT mRNA in TMZ-resistant clones compared to untreated cells. We also validated the data using Western blot analysis (Fig. 3C).

**Fig. 4:**
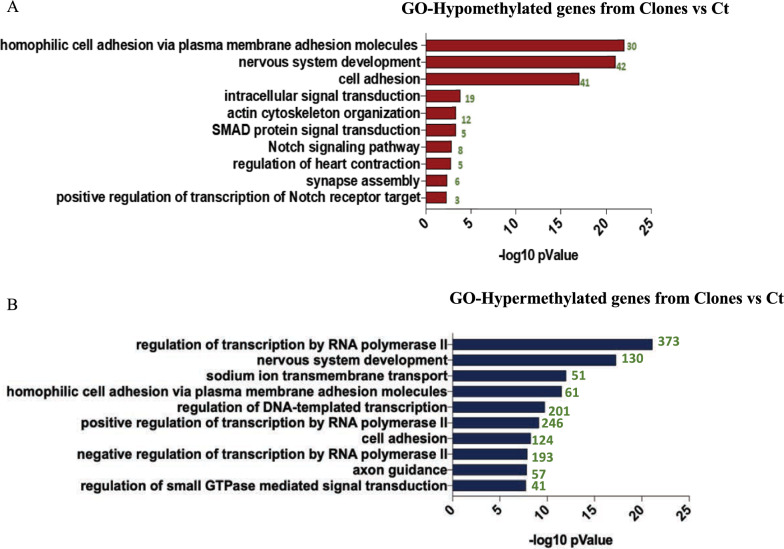
Gene Ontology of hypo- and hypermethylated genes from clones vs Cts: Gene Ontology (GO) plot representing the top ten biological pathways involving hypomethylated (**A**) and hypermethylated (**B**) genes comparing TMZ-resistant clones vs CT respectively. The -Log10 p-value is reported on X axis. The number of genes per pathway is reported in green

Given the large number of differentially methylated genes in TMZ-resistant clones, to narrow down the analysis and investigate the biological pathways in which the differentially methylated genes fall, we selected only genes that had at least 3 differentially methylated CpG sites between CT and TMZ-resistant clones. On this subset of genes, we performed gene ontology on hypo- and hypermethylated genes to evaluate their involvement in specific biological processes (Fig. 4A and B show the top ten enriched biological pathway). Differentially methylated regions enrichment was found in several biological pathways. We observed several genes hypermethylated in TMZ resistant clones belonging to the “regulation of transcription by RNA polymerase" pathway (Fig. 4A). In particular, we found hypermethylation in genes involved in mismatch repair (MMR) and base excision repair (BER), listed in Supplementary Table 1.

In conclusion, TMZ-resistant clones showed remarkable differences in methylation patterns compared to CT LN18 cell line. These differences are common to all clones, demonstrating that this epigenetic signature may be essential for establishing chemo-resistance regardless of glioblastoma tumor heterogeneity.

### Comparative analysis of epigenetic signatures in recurrent glioblastoma and temozolomide-resistant cell clones

To study whether the observed episignature acquired in TMZ-resistant clones is also present in recurrent glioblastomas, we analyzed methylation profiles in a longitudinal manner, in 4 patients affected by GB at first surgery and at recurrence after receiving TMZ as first chemotherapy treatment. All cases received a molecular diagnosis of glioblastoma IDH wild type. Tumor characterization of primary and recurrent tumors is shown in Table 1. First, we analyzed MGMT methylation status also in recurrent GB and we found a trend of hypermethylation in the gene body. In particular, we found a significant increase in methylation at CpG 15568398 located within the MGMT gene body. Methylation status of MGMT gene in patient cohort is shown in supplementary Fig. 1A. This feature was common to clones under pharmacological pressure. However, in TMZ-resistant clones, the effect is more widespread at many more CpG sites in the gene body, likely due to the more selective pressure exerted in vitro at high doses of TMZ and to different conditions between cell cultures and tumor micro-environment in vivo. Interestingly, when we analysed MGMT gene expression, extrapolated from an available dataset (GEO data repository), in a cohort of primitive and recurrent glioblastomas, we found a significant increase in MGMT expression in recurrent glioblastomas (Supplementary Fig. 1 B).

**Fig. 5:**
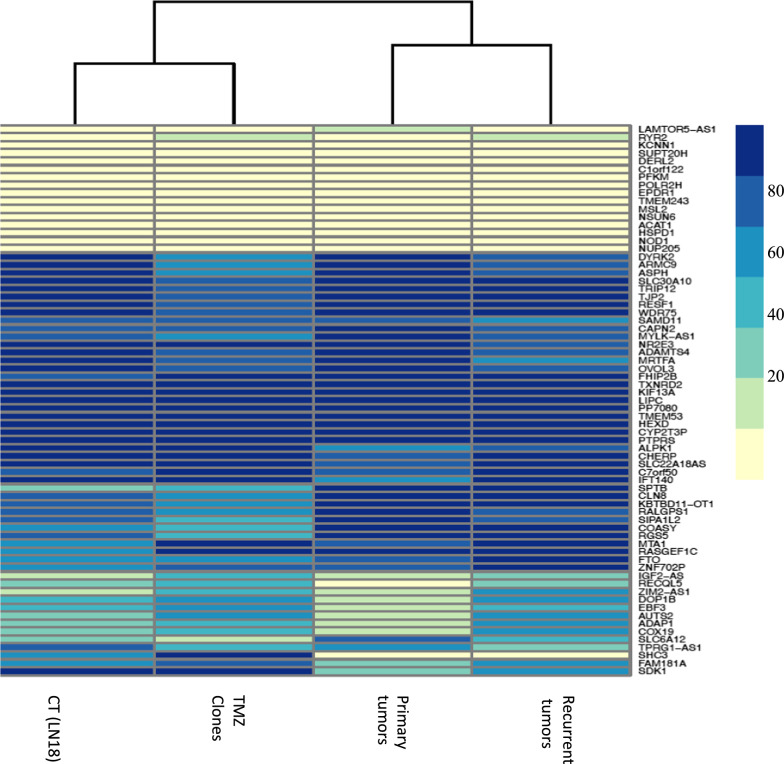
“TMZ epigenetic profiles” vs “Recurrence epigenetic profiles”: a cross analysis: Hierarchical Clustering Heatmap reporting the subset of genes (listed on the right) that are differentially methylated in both TMZ-resistant clones (clones vs CT) and Recurrences (recurrence vs primary tumors). As shown in figure, differential methylation values per genes are represented by associating a color scale to ß- values, from 0 (in light yellow) to 1 (in blue)

To investigate possible “episignatures” shared by TMZ-resistant clones and recurrent GB, we performed a cross analysis comparing differentially methylated genes in these two conditions. In the following analysis, we will refer to two “episignatures”: “TMZ epigenetic profile”, obtained from differentially methylated genes between CT and TMZ-resistant clones, and “recurrence epigenetic profile”, obtained from differentially methylated genes between primary and recurrent tumors. Both episignatures shared 53 common hypermethylated genes and 109 hypomethylated genes. The heat map shown in Fig. 5 reports a subset of differentially methylated genes in recurrent GB and in TMZ-resistant clones compared to primary tumor and untreated cells. The lists of all individual genes demethylated or hypermethylated under TMZ pressure in vivo and in vitro are shown in Supplementary Tables 2 and 3. Several genes were found to be hypermethylated and the potential loss of their expression could putatively contribute to TMZ resistance and tumor aggressiveness. For example, PRDM1 and MEF2B are hypermethylated in TMZ-resistant clones and in recurrent GBs. PRDM1 is a transcriptional repressor involved in the regulation of immune responses. Its potential downregulation due to hypermethylation could contribute to diminish the anti-tumor immune responses thus promoting tumor recurrence [25–27]. MEF2B is a member of the MEF2 family involved in muscle development and neuronal differentiation. Previous reports have suggested that hypermethylation of MEF2B may lead to transcriptional silencing and contribute to tumorigenesis [28]. In GBs, aberrant methylation of MEF2B may inhibit critical pathways that regulate cell proliferation and apoptosis, potentially leading to increased invasive capacity of tumor cells [28]. To verify if hypermethylation of MEF2B was associated with a decreased expression, we performed qPCR and western blot analyses for MEF2B in TMZ resistant clones. We found a significant decrease of MEF2B expression (Supplementary Fig. 2A and B).

**Fig. 6:**
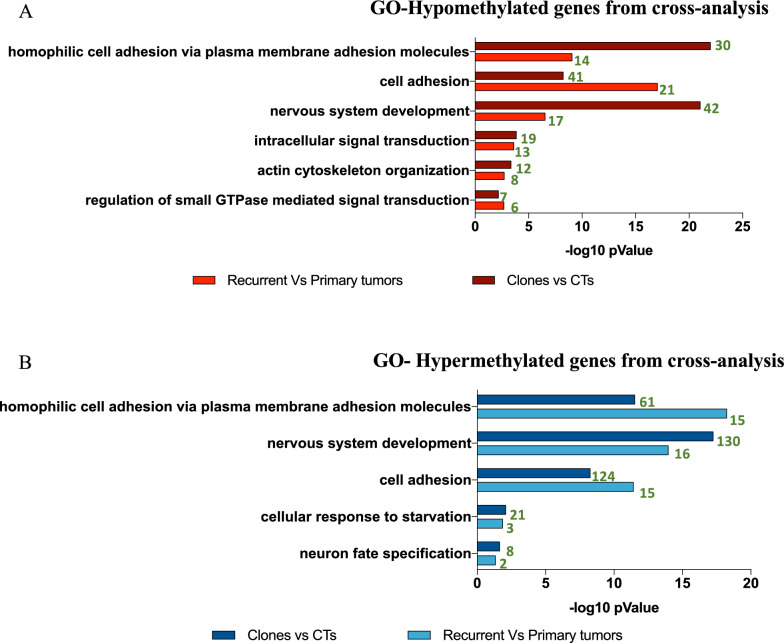
Gene Ontology of hypo- hypermethylated genes from TMZ vs Recurrent tumors epigenetic profiles. Gene Ontology (GO) plot representing the top ten biological pathway including hypomethylated (**A**) and hypermethylated (**B**) genes, comparing TMZ epigenetic profiles and recurrence epigenetic profiles. The-Log 10 p Value is reported on x axis. The number of genes per pathway is reported in green

At the same time, we have identified a number of genes whose hypomethylation, potentially associated with an increase in gene transcription, may be involved in tumor progression Interestingly, a hypomethylation in MAPK4/6 emerged [29]. (Supplementary Table 2). To verify if hypomethylation of MAPK6 was associated with an increase of gene expression we performed a qPCR and western blot analyses. We found a significant increase in mRNA and protein expression (Supplementary Fig. 2C and D). Finally, a comparative analysis between the gene ontology derived from TMZ epigenetic profiles and the gene ontology derived from recurrent GBs epigenetic profiles revealed several common pathways, as shown in Fig. 6.

## Discussion

The development of TMZ resistance is a crucial problem for glioblastoma patients. Numerous genes and pathways appear to be implicated in such onset [30, 31] In this manuscript we generated and characterized LN18 glioblastoma TMZ resistant cells. We analyzed the *episignatures* acquired during TMZ resistance development. First of all, we found a heterogenous status of MGMT promoter methylation. Indeed, we observed a loss of MGMT methylation in the majority of the TMZ-resistant clones by MSP. We also reported the non-statistically significant methylation levels in clones in the upstream regions from transcription start sites (TSS) by EPIC array. However, even if we observed a slight demethylation in clones (in the regions closer to TSS), we cannot observe concordance between MSP and EPIC array data, as the CpG sites investigated by the two techniques are different. Indeed, as previously described [21], MSP investigated the primary regulatory region of the MGMT gene, which is located downstream of the TSS.

We also found a significant increase in hypermethylation in MGMT gene body by EPIC array in clones. We evaluated if MGMT gene body hypermethylation correlated with MGMT gain of expression. Interestingly, we noticed a significant increase in MGMT gene expression that it was associated with an increase of MGMT protein levels. These results suggest that resistance to TMZ due to MGMT gene expression is associated with hypermethylation at the MGMT gene body, rather than promoter demethylation alone. This is an important, possible way to follow the development of drug resistance.

Global epigenomic profiling of TMZ resistant clones revealed widespread changes in methylation status with a strong increase in hypermethylation status suggesting a shift toward transcriptional repression at the epigenetic level. The analysis of hypermethylated genes in TMZ-resistant clones associated with potential loss of gene expression showed an increase in various biological pathways. For example, we found hypermethylation of “genes involved in the regulation of transcription by RNA polymerase", including genes involved in MMR response. Hypermethylation of the MSH5 gene has been investigated in recurrent glioblastoma [32]. Several studies suggest that alterations in the methylation status of DNA repair genes may influence tumor biology and response to therapies such as chemotherapy and radiation [19]. For example, there is evidence that methylation of genes involved in mismatch repair, including MSH5, is associated with altered therapeutic response, with hypermethylation potentially impeding DNA repair functions and facilitating tumor progression [19]. For these reasons, the epigenetic alterations of MMR genes could have an impact on TMZ resistance. Consequently, targeting of MMR system could help to hinder TMZ resistance; However, further studies are needed at this stage to demonstrate its real implications for the development of drug resistance. To evaluate common epigenetic signatures in recurrent glioblastomas, which received Stupp protocol (TMZ plus radiotherapy), and TMZ resistant clones obtained in vitro, we compared the methylation profiles of recurrent glioblastoma samples and epigenetic profile of TMZ resistant clones. We found that many of the epigenetic alterations observed in TMZ-resistant clones were also present in recurrent tumors. Specifically, we identified 53 hypermethylated genes and 109 hypomethylated genes shared between TMZ-resistant clones and recurrent tumors. This suggests that epigenetic changes acquired during TMZ treatment may be a shared mechanism of resistance. Hypermethylation of genes such as MEF2B, involved in neuronal differentiation, could contribute to increased tumor invasiveness. Additionally, hypomethylation of MAPK4/6 in recurrent tumors has been associated with more aggressive cellular behavior, supporting the idea that epigenetic changes in TMZ-resistant cells create a permissive environment for tumor growth and progression [29]. Recent studies have shown that hypomethylation of the MAPK4/6 gene correlates with high levels of MAPK4/6 protein, which in turn facilitates aggressive cellular behavior through pathways such as AKT/mTOR, promoting cell proliferation and survival [33]. This supports the hypothesis that a hypomethylated state of MAPK4/6 creates a permissive environment for GB cell growth and resistance to TMZ [33]. Our data suggested that the development of TMZ resistance involve several molecular alterations. I*n primis*, the methylation status of the MGMT gene [34] but there are cases of GBs resistant to TMZ treatment regardless of the methylation status of MGMT. Thus, alterations in other pathways could occur in the development of TMZ resistance [35]. However, further studies are necessary to demonstrate the role of these gene alterations in TMZ development and if the targeting of these genes could help to reduce TMZ resistance.

## Conclusions

Altogether our data suggest that TMZ induces significant changes in the epigenomic landscape, both in vitro cellular models and in vivo recurrent GB. These epigenetic alterations may be consistent with TMZ-resistance and increased aggressiveness of GB observed in recurrent tumors.

However, a limitation of this study is the relatively small number of longitudinal patient cohorts analyzed. Nevertheless, our findings strongly encourage further investigation of DNA methylation alterations associated with TMZ resistance. It would be particularly important to translate the model of temozolomide resistance from glioblastoma cell lines to primary tumor cell lines cultured at the time of initial surgical resection and subjected to selective pressure with TMZ. These TMZ-resistant glioblastoma cells provide a valuable opportunity to study epigenetic alterations in tumor recurrence and may provide insights into precision medicine strategies to overcome chemoresistance.

## Supplementary Information

Supplementary material 1: Figure 1. CpG methylation differences in MGMT gene body and TSS upstream region: Primary vs recurrent tumors A) Schematic representation of the MGMT gene. Upstream regions from transcription start sites (TSS), specifically 200 bp and 1500 bp, are represented in green (TSS200-1500). Exons are represented in blue (ex), and introns are represented in light blue (intr). The TSS is shown as a red bar. Exon 1 is shown in light blue due to its regulatory role in MGMT transcription. The green, orange, yellow and pink rectangles represent different regions showing changes in DNA methylation within the gene. The CpG coordinates are derived from the hg38 genome. The average methylation per CpG site, with its standard error, is reported. The values detected in primary tumors are represented in green (reported as “GB p”), whereas the values reported in recurrent tumors are represented in red (reported as “GB r”). The sole statistically significant, differentially methylated CpG is indicated (*p<0.05). B) Boxplot of MGMT expression levels in primitive GB vs recurrent GB (*p= 0.02228).

Supplementary material 2: Figure 2. MEF2B and MAPK6 mRNA and protein levels in Clones vs Cts A) mRNA expression levels of MEF2B and B) MAPK6 were assessed by qPCR. Statistical analyses were performed using one-way ANOVA following by multiple *t-test* analysis. All values are given as the mean ± standard deviation of at least three replicates (* p-value summary = 0.003; *p-value summary= 0.05). C) LN18 cells (treated with DMSO) and TMZ-resistant LN18 clones were collected and lysed. The extracted proteins were separated by SDS-PAGE, blotted, and probed for MEF2B and MAPK6. Protein levels were normalized by probing for α-tubulin. The molecular weight indicated by the protein ladder was reported.

Supplementary material 3.

Supplementary material 4.

Supplementary material 5.

Supplementary material 6.

## Data Availability

The original data applied in this research are availed from the corresponding authors.

## Abbreviations

GB: Glioblastoma
CT: Controls
C: Clones
MGMT: O6-Methylguanine-DNA Methyltransferase
TMZ: Temozolomide
MMR: Mismatch repair
BER: Base-excision repair
GO: Gene ontology
MSP: Methylation-specific PCR
PCA: Principal component analysis
TSS: Transcription start site
GEO: Gene expression omnibus
CNS: Central nervous system
