# Supplementary figures and images for: DNA methylation remodeling in temozolomide resistant recurrent glioblastoma: comparing epigenetic dynamics in vitro and in vivo
Source: J Transl Med. 2025 Jul 10;23:779. doi: 10.1186/s12967-025-06767-x (PMC12247454; doi:10.1186/s12967-025-06767-x)

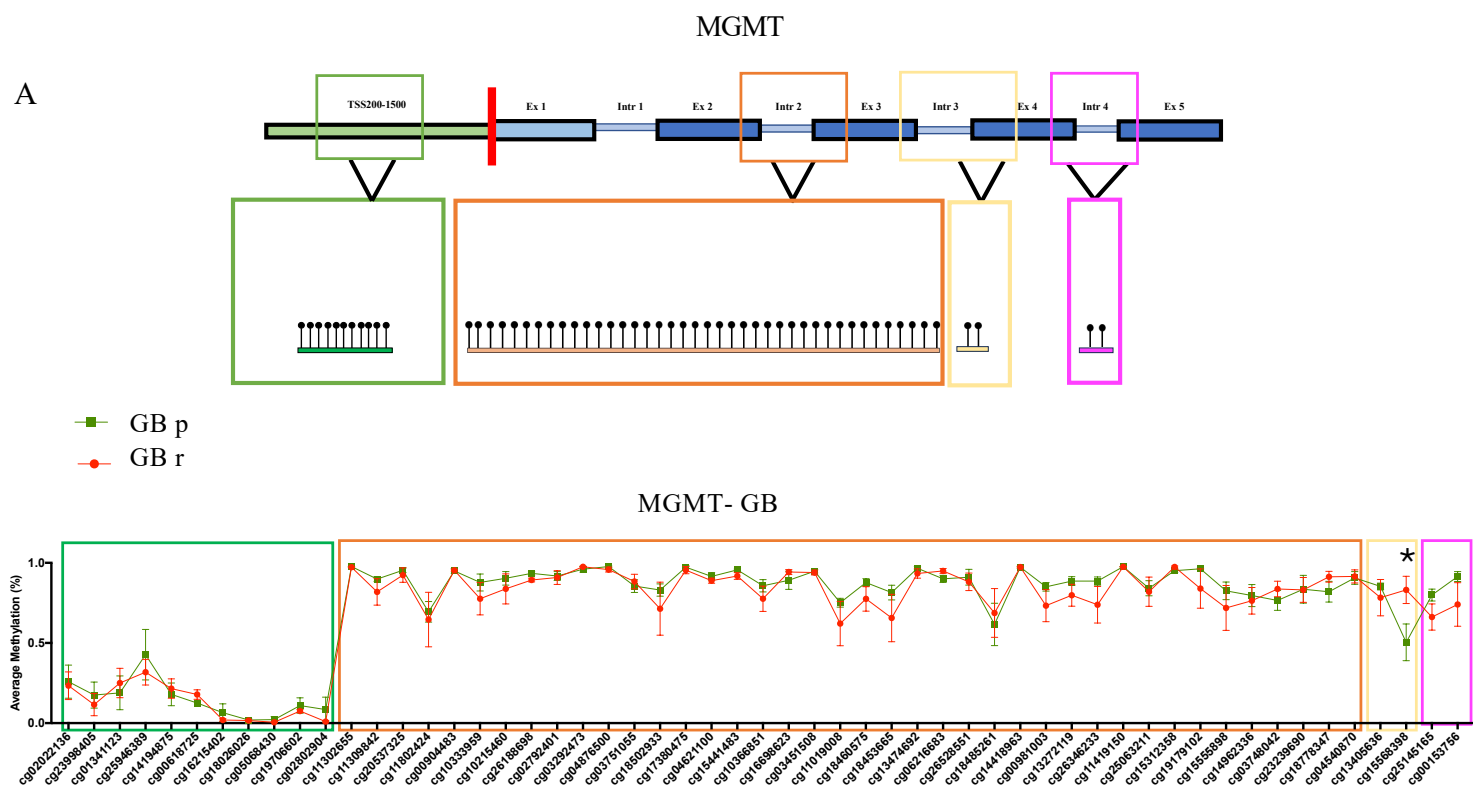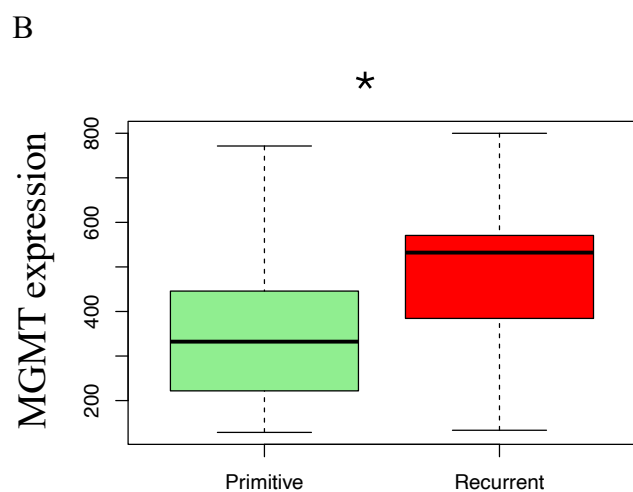

Supplement: Supplementary file 1 — Supplementary material 1: Figure 1. CpG methylation differences in MGMT gene body and TSS upstream region: Primary vs recurrent tumors A) Schematic representation of the MGMT gene. Upstream regions from transcription start sites (TSS), specifically 200 bp and 1500 bp, are represented in green (TSS200-1500). Exons are represented in blue (ex), and introns are represented in light blue (intr). The TSS is shown as a red bar. Exon 1 is shown in light blue due to its regulatory role in MGMT transcription. The green, orange, yellow and pink rectangles represent different regions showing changes in DNA methylation within the gene. The CpG coordinates are derived from the hg38 genome. The average methylation per CpG site, with its standard error, is reported. The values detected in primary tumors are represented in green (reported as “GB p”), whereas the values reported in recurrent tumors are represented in red (reported as “GB r”). The sole statistically significant, differentially methylated CpG is indicated (*p<0.05). B) Boxplot of MGMT expression levels in primitive GB vs recurrent GB (*p= 0.02228). [file 12967_2025_6767_MOESM1_ESM.pdf]

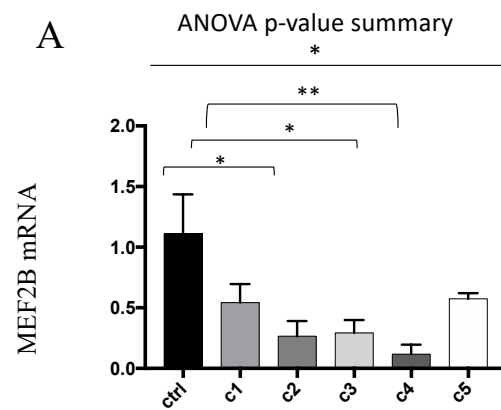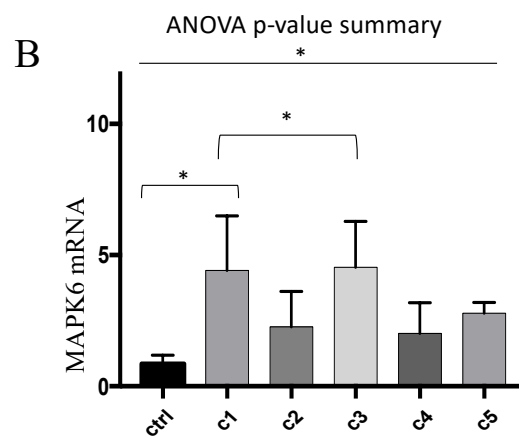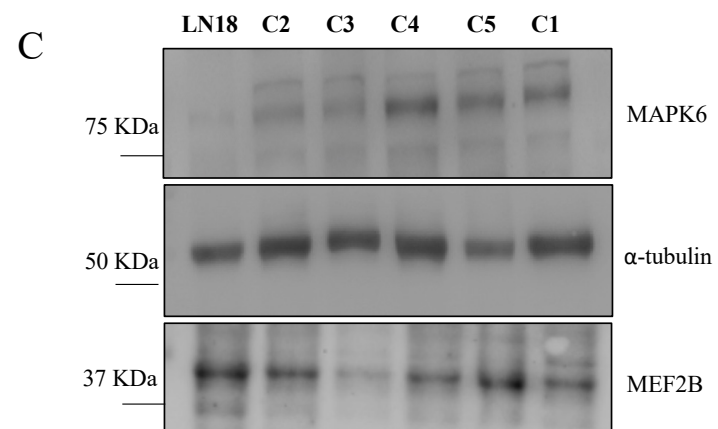

Supplement: Supplementary file 2 — Supplementary material 2: Figure 2. MEF2B and MAPK6 mRNA and protein levels in Clones vs Cts A) mRNA expression levels of MEF2B and B) MAPK6 were assessed by qPCR. Statistical analyses were performed using one-way ANOVA following by multiple t-test analysis. All values are given as the mean ± standard deviation of at least three replicates (* p-value summary = 0.003; *p-value summary= 0.05). C) LN18 cells (treated with DMSO) and TMZ-resistant LN18 clones were collected and lysed. The extracted proteins were separated by SDS-PAGE, blotted, and probed for MEF2B and MAPK6. Protein levels were normalized by probing for α-tubulin. The molecular weight indicated by the protein ladder was reported. [file 12967_2025_6767_MOESM2_ESM.pdf]
