# Supplementary material for: DNA methylation remodeling in temozolomide resistant recurrent glioblastoma: comparing epigenetic dynamics in vitro and in vivo
Source: J Transl Med. 2025 Jul 10;23:779. doi: 10.1186/s12967-025-06767-x (PMC12247454; doi:10.1186/s12967-025-06767-x)
Supplement: Supplementary file 4 — Supplementary material 4. [file 12967_2025_6767_MOESM4_ESM.docx]

**Table 1.** DNA methylation levels at differentially methylated CpG sites significantly less methylated in both Resistant Clones and Recurrent GB.

| **CpG ID** | **Gene Name** | **Chromosome** | **Start** | **End** | **DNA Methylation (%) in Untreated Clones** | **DNA Methylation (%) in Resistant Clones** | **DNA methylation (%) in Primitive GB** | **DNA methylation (%) in Recurrent GB** |
| --- | --- | --- | --- | --- | --- | --- | --- | --- |
| cg26835636 | ABCB1 | 7 | 87628474 | 87628475 | 95,71% | 91,40% | 10,67% | 2,13% |
| cg14994056 | ACAT1 | 11 | 108121288 | 108121289 | 2,09% | 1,06% | 8,71% | 2,22% |
| cg15889722 | ADAMTS4 | 1 | 161196624 | 161196625 | 86,45% | 76,66% | 93,54% | 73,34% |
| cg00402074 | ADGRF5 | 6 | 46922005 | 46922006 | 72,01% | 57,28% | 93,71% | 81,76% |
| cg19917507 | ALPK2 | 18 | 58541723 | 58541724 | 5,37% | 2,64% | 96,60% | 89,59% |
| cg13313836 | ANXA2 | 15 | 60395085 | 60395086 | 46,85% | 15,89% | 87,87% | 73,15% |
| cg15119597 | AQP4-AS1 | 18 | 26777940 | 26777941 | 13,56% | 7,82% | 93,52% | 83,72% |
| cg16090476 | ARHGAP24 | 4 | 85953888 | 85953889 | 10,38% | 1,79% | 96,84% | 89,29% |
| cg11558318 | ARHGAP35 | 19 | 46951874 | 46951875 | 50,10% | 31,52% | 92,38% | 82,98% |
| cg14900199 | ARMC9 | 2 | 231222371 | 231222372 | 90,01% | 67,17% | 94,23% | 79,86% |
| cg00168967 | ASPH | 8 | 61593001 | 61593002 | 87,62% | 65,96% | 90,81% | 81,04% |
| cg01255059 | BACH1 | 21 | 29221701 | 29221702 | 8,37% | 3,23% | 96,12% | 87,10% |
| cg02130836 | BHMT2 | 5 | 79074832 | 79074833 | 2,03% | 0,98% | 84,08% | 65,46% |
| cg24828792 | BMP1 | 8 | 22169666 | 22169667 | 24,44% | 14,35% | 93,24% | 72,80% |
| cg04987149 | C1orf122 | 1 | 37807712 | 37807713 | 1,14% | 0,33% | 0,73% | 0,29% |
| cg24200259 | CACNG2 | 22 | 36702414 | 36702415 | 10,24% | 1,22% | 65,70% | 34,17% |
| cg05125455 | CADM1 | 11 | 115198425 | 115198426 | 3,03% | 1,50% | 95,13% | 87,35% |
| cg22828602 | CADPS2 | 7 | 122698525 | 122698526 | 7,86% | 3,51% | 94,31% | 86,08% |
| cg06740995 | CAPN2 | 1 | 223705739 | 223705740 | 80,33% | 71,44% | 95,23% | 73,05% |
| cg10266547 | CD81-AS1 | 11 | 2344723 | 2344724 | 23,74% | 13,61% | 97,59% | 94,52% |
| cg21142548 | CDKN1B | 12 | 12716464 | 12716465 | 2,84% | 0,77% | 22,03% | 5,89% |
| cg14076497 | CLN8 | 8 | 1779563 | 1779564 | 79,04% | 60,95% | 95,57% | 87,05% |
| cg15138339 | COASY | 17 | 42563204 | 42563205 | 63,24% | 51,63% | 96,20% | 85,70% |
| cg04583448 | CPQ | 8 | 96853644 | 96853645 | 13,03% | 6,25% | 88,00% | 75,52% |
| cg21735068 | CPQ | 8 | 96963239 | 96963240 | 2,73% | 1,55% | 68,03% | 34,30% |
| cg07001481 | DERL2 | 17 | 5486349 | 5486350 | 0,48% | 0,19% | 0,51% | 0,15% |
| cg12183518 | DGKG | 3 | 186283782 | 186283783 | 16,97% | 10,49% | 92,14% | 75,39% |
| cg05810341 | DLGAP2 | 8 | 1093787 | 1093788 | 8,93% | 5,45% | 93,40% | 74,93% |
| cg27276522 | DYRK2 | 12 | 67655745 | 67655746 | 85,56% | 70,20% | 92,64% | 80,84% |
| cg09064095 | ECHDC3 | 10 | 11745030 | 11745031 | 13,68% | 5,98% | 93,42% | 74,71% |
| cg18686797 | ELAVL4 | 1 | 50105818 | 50105819 | 27,04% | 9,46% | 94,93% | 84,53% |
| cg25783719 | EPDR1 | 7 | 37916832 | 37916833 | 2,77% | 0,71% | 1,00% | 0,30% |
| cg13368214 | ERCC1 | 19 | 45449344 | 45449345 | 64,40% | 50,69% | 95,99% | 79,48% |
| cg21793795 | EYA2 | 20 | 47124529 | 47124530 | 17,21% | 7,66% | 82,38% | 57,52% |
| cg08418079 | GALNT18 | 11 | 11426570 | 11426571 | 20,53% | 6,55% | 93,27% | 86,23% |
| cg08652333 | HECW1 | 7 | 43558038 | 43558039 | 49,13% | 36,38% | 92,19% | 74,18% |
| cg13162810 | HSPD1 | 2 | 197515473 | 197515474 | 4,09% | 1,34% | 6,39% | 1,31% |
| cg21596294 | IL33 | 9 | 6247616 | 6247617 | 3,53% | 1,17% | 94,57% | 84,73% |
| cg18208851 | ITPR3 | 6 | 33623205 | 33623206 | 28,48% | 13,05% | 91,10% | 78,66% |
| cg09975553 | JAKMIP2-AS1 | 5 | 147617208 | 147617209 | 2,19% | 0,99% | 97,47% | 94,15% |
| cg14076497 | KBTBD11-OT1 | 8 | 1779563 | 1779564 | 79,04% | 60,95% | 95,57% | 87,05% |
| cg14433357 | KCNN1 | 19 | 17951322 | 17951323 | 11,28% | 3,83% | 5,54% | 2,16% |
| cg19852660 | KCNQ1 | 11 | 2825451 | 2825452 | 2,71% | 1,15% | 72,86% | 46,12% |
| cg05874233 | KIF13A | 6 | 17973430 | 17973431 | 96,92% | 92,26% | 96,66% | 90,85% |
| cg20982476 | LAMTOR5-AS1 | 1 | 110408149 | 110408150 | 11,47% | 5,12% | 20,11% | 3,71% |
| cg01255059 | LINC00189 | 21 | 29221701 | 29221702 | 8,37% | 3,23% | 96,12% | 87,10% |
| cg23101649 | LINC00551 | 13 | 106702253 | 106702254 | 3,39% | 0,87% | 93,30% | 79,49% |
| cg02071598 | LINC01091 | 4 | 123574454 | 123574455 | 49,15% | 37,07% | 94,30% | 79,77% |
| cg15336255 | LINC01091 | 4 | 123649945 | 123649946 | 0,69% | 0,43% | 92,66% | 66,01% |
| cg23385898 | LINC01117 | 2 | 176596124 | 176596125 | 49,20% | 36,27% | 95,79% | 89,18% |
| cg09243811 | LINC01482 | 17 | 68698445 | 68698446 | 24,46% | 8,09% | 78,82% | 47,47% |
| cg07689590 | LINC01491 | 15 | 47846016 | 47846017 | 3,47% | 0,94% | 92,34% | 70,75% |
| cg08997126 | LINC01681 | 1 | 170200835 | 170200836 | 19,62% | 4,21% | 80,98% | 63,17% |
| cg22663660 | LIPC | 15 | 58440555 | 58440556 | 97,03% | 94,66% | 96,98% | 91,23% |
| cg13888588 | LTBP1 | 2 | 33012160 | 33012161 | 45,66% | 33,04% | 82,38% | 62,53% |
| cg07596136 | MACROD2 | 20 | 15022888 | 15022889 | 17,83% | 7,07% | 83,97% | 69,03% |
| cg18300998 | MAPK6 | 15 | 52031707 | 52031708 | 19,96% | 8,55% | 88,81% | 72,43% |
| cg11622428 | MED12L | 3 | 151241054 | 151241055 | 10,72% | 4,15% | 96,38% | 90,94% |
| cg24805739 | MED13L | 12 | 116152161 | 116152162 | 68,80% | 55,12% | 94,85% | 88,68% |
| cg00632489 | MICAL2 | 11 | 12160830 | 12160831 | 70,75% | 51,21% | 93,92% | 83,80% |
| cg03044494 | MIR100HG | 11 | 122089019 | 122089020 | 68,36% | 47,54% | 92,35% | 81,07% |
| cg24034459 | MIR4713HG | 15 | 51056702 | 51056703 | 4,93% | 1,67% | 90,81% | 70,43% |
| cg16224128 | MRTFA | 22 | 40603147 | 40603148 | 90,04% | 80,63% | 89,81% | 64,99% |
| cg25961579 | MSL2 | 3 | 136197121 | 136197122 | 1,93% | 0,82% | 2,15% | 0,83% |
| cg00651277 | MYLK-AS1 | 3 | 123636422 | 123636423 | 79,99% | 66,96% | 92,16% | 71,84% |
| cg00248242 | NDUFA10 | 2 | 239927642 | 239927643 | 2,39% | 1,08% | 96,06% | 89,03% |
| cg20988238 | NECTIN4 | 1 | 161084348 | 161084349 | 45,08% | 28,43% | 96,26% | 88,58% |
| cg16001865 | NFIA | 1 | 61171050 | 61171051 | 56,40% | 33,71% | 91,92% | 80,06% |
| cg17946043 | NOD1 | 7 | 30478629 | 30478630 | 3,03% | 1,65% | 4,32% | 1,66% |
| cg27448447 | NR2E3 | 15 | 71801009 | 71801010 | 94,64% | 89,33% | 88,57% | 72,98% |
| cg18012811 | NSUN6 | 10 | 18651835 | 18651836 | 1,47% | 0,59% | 2,37% | 0,80% |
| cg15472802 | NUP205 | 7 | 135558721 | 135558722 | 3,94% | 1,95% | 5,11% | 2,27% |
| cg05900441 | OVOL3 | 19 | 36111643 | 36111644 | 89,08% | 81,41% | 88,46% | 72,04% |
| cg07013942 | P3H2 | 3 | 190032114 | 190032115 | 3,92% | 2,01% | 80,65% | 59,49% |
| cg17901924 | PCNX2 | 1 | 232988672 | 232988673 | 8,76% | 4,48% | 88,90% | 74,71% |
| cg26946232 | PDE10A | 6 | 165676824 | 165676825 | 4,31% | 1,20% | 96,49% | 91,24% |
| cg06098346 | PFKM | 12 | 48105884 | 48105885 | 0,74% | 0,27% | 0,86% | 0,22% |
| cg20979815 | PIWIL4 | 11 | 94563741 | 94563742 | 20,73% | 10,42% | 82,45% | 53,31% |
| cg12942878 | PLAAT1 | 3 | 193242843 | 193242844 | 1,21% | 0,58% | 93,10% | 58,56% |
| cg02009228 | PLEKHG4B | 5 | 168356 | 168357 | 67,61% | 53,11% | 91,69% | 83,00% |
| cg07892496 | POLR2H | 3 | 184362876 | 184362877 | 0,67% | 0,31% | 1,04% | 0,40% |
| cg07234599 | RALGPS1 | 9 | 127065887 | 127065888 | 77,06% | 57,82% | 94,97% | 77,55% |
| cg18174702 | RESF1 | 12 | 31990240 | 31990241 | 89,67% | 82,82% | 96,12% | 88,42% |
| cg16793817 | RGS5 | 1 | 163171133 | 163171134 | 77,29% | 49,85% | 96,56% | 89,94% |
| cg11577089 | RMDN2 | 2 | 38037582 | 38037583 | 19,76% | 6,41% | 89,54% | 77,49% |
| cg04153193 | RMDN2-AS1 | 2 | 38037820 | 38037821 | 31,64% | 12,19% | 91,82% | 68,83% |
| cg22828602 | RNF133 | 7 | 122698525 | 122698526 | 7,86% | 3,51% | 94,31% | 86,08% |
| cg07596136 | RNU6-115P | 20 | 15022888 | 15022889 | 17,83% | 7,07% | 83,97% | 69,03% |
| cg15334482 | ROR1 | 1 | 64112459 | 64112460 | 8,87% | 3,85% | 90,83% | 75,92% |
| cg10266547 | RPL26P30 | 11 | 2344723 | 2344724 | 23,74% | 13,61% | 97,59% | 94,52% |
| cg09756115 | SAMD11 | 1 | 944035 | 944036 | 81,27% | 71,55% | 84,52% | 66,20% |
| cg00090697 | SDC2 | 8 | 96511181 | 96511182 | 12,58% | 3,25% | 96,65% | 91,79% |
| cg23356017 | SDC2 | 8 | 96498331 | 96498332 | 7,47% | 2,87% | 94,18% | 83,90% |
| cg06727055 | SESN1 | 6 | 109082075 | 109082076 | 21,73% | 9,96% | 93,51% | 79,97% |
| cg24066742 | SEZ6L | 22 | 26347569 | 26347570 | 1,08% | 0,43% | 95,30% | 89,16% |
| cg09132058 | SIPA1L2 | 1 | 232548367 | 232548368 | 75,15% | 56,45% | 95,99% | 82,69% |
| cg02834165 | SLC30A10 | 1 | 219793103 | 219793104 | 86,64% | 71,78% | 95,71% | 85,67% |
| cg09231580 | SLC6A12 | 12 | 212039 | 212040 | 39,56% | 25,90% | 77,80% | 56,23% |
| cg14647872 | SMAD7 | 18 | 48943177 | 48943178 | 12,68% | 4,85% | 88,74% | 64,62% |
| cg02733067 | SPECC1 | 17 | 20090768 | 20090769 | 58,53% | 44,82% | 96,02% | 86,89% |
| cg11862917 | SSBP3 | 1 | 54249877 | 54249878 | 45,29% | 25,68% | 97,55% | 93,73% |
| cg06035369 | SUPT20H | 13 | 37059411 | 37059412 | 0,65% | 0,34% | 1,51% | 0,56% |
| cg01952553 | SV2C | 5 | 76204782 | 76204783 | 3,89% | 1,92% | 92,98% | 81,35% |
| cg06235115 | TC2N | 14 | 91858482 | 91858483 | 29,02% | 15,97% | 93,27% | 84,24% |
| cg24647271 | TEKT1 | 17 | 6815362 | 6815363 | 13,94% | 4,17% | 73,80% | 40,64% |
| cg25918166 | TEX41 | 2 | 144944246 | 144944247 | 60,59% | 45,38% | 95,10% | 86,42% |
| cg19942994 | TJP2 | 9 | 69176840 | 69176841 | 92,90% | 75,99% | 96,58% | 92,06% |
| cg11256279 | TMEM243 | 7 | 87219987 | 87219988 | 2,31% | 1,26% | 3,01% | 0,92% |
| cg15932455 | TMTC1 | 12 | 29566465 | 29566466 | 9,23% | 5,10% | 97,18% | 92,89% |
| cg03176993 | TPRG1-AS1 | 3 | 188947098 | 188947099 | 72,95% | 54,72% | 64,43% | 35,62% |
| cg24671197 | TRIP12 | 2 | 229835557 | 229835558 | 86,30% | 75,00% | 96,46% | 89,34% |
| cg00179699 | TSHZ2 | 20 | 53412965 | 53412966 | 6,32% | 2,39% | 77,94% | 43,06% |
| cg02451548 | TSPAN14 | 10 | 80478042 | 80478043 | 48,51% | 31,34% | 92,12% | 78,76% |
| cg01057705 | TXNRD2 | 22 | 19905143 | 19905144 | 93,71% | 87,75% | 96,63% | 90,74% |
| cg14557690 | UAP1L1 | 9 | 137083841 | 137083842 | 3,44% | 0,85% | 88,18% | 71,89% |
| cg23068989 | UBE2U | 1 | 64223745 | 64223746 | 36,11% | 24,89% | 97,24% | 90,63% |
| cg06111946 | VAC14 | 16 | 70713936 | 70713937 | 65,30% | 45,78% | 94,55% | 75,51% |
| cg20764815 | VAV3 | 1 | 107627190 | 107627191 | 28,09% | 11,43% | 95,40% | 83,43% |
| cg19103456 | VAV3 | 1 | 107649147 | 107649148 | 60,00% | 43,99% | 92,41% | 80,55% |
| cg22039204 | VCAN-AS1 | 5 | 83559749 | 83559750 | 48,44% | 29,32% | 94,73% | 86,59% |
| cg09775648 | WDR75 | 2 | 189444460 | 189444461 | 92,66% | 80,54% | 95,39% | 85,05% |
| cg24751865 | ZNF133 | 20 | 18315111 | 18315112 | 19,25% | 5,14% | 96,98% | 83,45% |
