# Supplementary material for: DNA methylation remodeling in temozolomide resistant recurrent glioblastoma: comparing epigenetic dynamics in vitro and in vivo
Source: J Transl Med. 2025 Jul 10;23:779. doi: 10.1186/s12967-025-06767-x (PMC12247454; doi:10.1186/s12967-025-06767-x)
Supplement: Supplementary file 5 — Supplementary material 5. [file 12967_2025_6767_MOESM5_ESM.docx]

**Table 1.** DNA methylation levels at differentially methylated CpG sites significantly more methylated in both Resistant Clones and Recurrent GB.

| **CpG ID** | **Gene Name** | **Chromosome** | **Start** | **End** | **DNA Methylation (%) in Untreated Clones** | **DNA Methylation (%) in Resistant Clones** | **DNA methylation (%) in Primitive GB** | **DNA methylation (%) in Recurrent GB** |
| --- | --- | --- | --- | --- | --- | --- | --- | --- |
| cg05528492 | ADAP1 | 7 | 907020 | 907021 | 41.19% | 53.06% | 27.63% | 59.89% |
| cg06822690 | ADGRL2 | 1 | 81574324 | 81574325 | 1.79% | 11.59% | 11.19% | 26.12% |
| cg07034362 | ALDH1L1 | 3 | 126180709 | 126180710 | 12.36% | 36.97% | 0.31% | 1.42% |
| cg02090418 | ALPK1 | 4 | 112412113 | 112412114 | 88.64% | 94.31% | 57.91% | 82.51% |
| cg12403745 | AUTS2 | 7 | 70776534 | 70776535 | 31.31% | 57.93% | 27.03% | 57.10% |
| cg24446071 | MEF2B | 19 | 19170461 | 19170462 | 93.60% | 96.77% | 6.16% | 34.60% |
| cg12076878 | C7orf50 | 7 | 1009913 | 1009914 | 83.06% | 90.70% | 72.22% | 92.17% |
| cg22344830 | CFAP74 | 1 | 2003609 | 2003610 | 19.73% | 31.93% | 3.49% | 12.70% |
| cg04390110 | CHERP | 19 | 16535517 | 16535518 | 94.04% | 96.85% | 73.66% | 96.49% |
| cg05528492 | COX19 | 7 | 907020 | 907021 | 41.19% | 53.06% | 27.63% | 59.89% |
| cg06859321 | CYP2T3P | 19 | 41137294 | 41137295 | 93.31% | 96.05% | 88.52% | 97.57% |
| cg11708406 | DOP1B | 21 | 36183496 | 36183497 | 50.28% | 63.81% | 24.05% | 56.90% |
| cg05636467 | EBF3 | 10 | 129871142 | 129871143 | 44.21% | 60.49% | 24.25% | 51.45% |
| cg26025596 | ESRRB | 14 | 76456317 | 76456318 | 9.45% | 16.47% | 54.93% | 82.21% |
| cg12173661 | FAM181A | 14 | 93926586 | 93926587 | 63.52% | 77.23% | 37.34% | 69.01% |
| cg10087374 | FHIP2B | 8 | 22102945 | 22102946 | 82.12% | 92.22% | 88.24% | 95.92% |
| cg02889177 | FNDC3B | 3 | 172153422 | 172153423 | 0.67% | 1.27% | 2.04% | 14.77% |
| cg12016828 | FTO | 16 | 53998132 | 53998133 | 56.68% | 67.09% | 84.66% | 95.49% |
| cg20834081 | GPC6 | 13 | 94157799 | 94157800 | 4.07% | 15.32% | 26.24% | 47.35% |
| cg13652281 | HEXD | 17 | 82435628 | 82435629 | 95.18% | 98.29% | 88.08% | 97.76% |
| cg24095592 | HOXB-AS3 | 17 | 48555840 | 48555841 | 88.57% | 93.73% | 2.04% | 9.57% |
| cg00209206 | IFT140 | 16 | 1524815 | 1524816 | 87.69% | 94.19% | 63.21% | 97.06% |
| cg06460568 | IGF2-AS | 11 | 2140981 | 2140982 | 21.53% | 44.96% | 15.18% | 32.72% |
| cg15144068 | IGHM | 14 | 105855646 | 105855647 | 2.20% | 9.61% | 59.77% | 84.57% |
| cg14725580 | ILRUN | 6 | 34695378 | 34695379 | 18.78% | 33.20% | 6.85% | 16.32% |
| cg00894289 | LMO2 | 11 | 33869371 | 33869372 | 11.51% | 21.23% | 0.79% | 2.64% |
| cg01780596 | LRRC73 | 6 | 43510530 | 43510531 | 1.72% | 2.79% | 4.83% | 12.65% |
| cg21169914 | MARCHF1 | 4 | 164383336 | 164383337 | 3.95% | 21.04% | 4.14% | 9.57% |
| cg15568398 | MGMT | 10 | 129758561 | 129758562 | 17.94% | 37.33% | 50.41% | 83.11% |
| cg18388431 | MTA1 | 14 | 105458080 | 105458081 | 68.65% | 85.05% | 75.52% | 92.40% |
| cg25893964 | MYO1D | 17 | 32780760 | 32780761 | 1.31% | 8.85% | 82.63% | 95.28% |
| cg11991859 | NPAS3 | 14 | 32939321 | 32939322 | 0.45% | 0.96% | 1.08% | 3.94% |
| cg03411548 | PNPLA6 | 19 | 7536121 | 7536122 | 25.24% | 41.17% | 2.82% | 9.53% |
| cg14808378 | PP7080 | 5 | 466467 | 466468 | 96.81% | 98.68% | 94.79% | 98.24% |
| cg19577529 | PRDM1 | 6 | 106097792 | 106097793 | 6.99% | 28.86% | 2.25% | 8.87% |
| cg23864120 | PTPRS | 19 | 5239040 | 5239041 | 92.10% | 96.19% | 87.66% | 96.48% |
| cg16150702 | RASGEF1C | 5 | 180127593 | 180127594 | 69.69% | 90.05% | 85.40% | 96.04% |
| cg12691994 | RASSF5 | 1 | 206575523 | 206575524 | 87.04% | 93.20% | 4.29% | 25.14% |
| cg21979250 | RECQL5 | 17 | 75654285 | 75654286 | 41.85% | 55.28% | 8.83% | 28.61% |
| cg01968657 | RYR2 | 1 | 237043956 | 237043957 | 3.52% | 16.83% | 5.41% | 14.82% |
| cg19230787 | SDK1 | 7 | 4010335 | 4010336 | 89.38% | 94.15% | 38.41% | 65.95% |
| cg14048780 | SHC3 | 9 | 89178450 | 89178451 | 64.40% | 86.81% | 0.15% | 0.65% |
| cg12563184 | SLC22A18AS | 11 | 2901261 | 2901262 | 94.36% | 96.70% | 75.82% | 91.33% |
| cg23241781 | SLC8A3 | 14 | 70187247 | 70187248 | 61.21% | 93.27% | 0.97% | 6.47% |
| cg13101284 | SLIT3 | 5 | 169174549 | 169174550 | 1.36% | 8.42% | 57.08% | 83.71% |
| cg19956971 | SMG6 | 17 | 2179826 | 2179827 | 30.75% | 51.42% | 6.98% | 20.27% |
| cg17956145 | SNX29 | 16 | 12147959 | 12147960 | 51.21% | 70.37% | 2.75% | 10.94% |
| cg03731303 | SOX2-OT | 3 | 181725221 | 181725222 | 12.32% | 26.49% | 4.35% | 19.52% |
| cg00114569 | SPTB | 14 | 64772609 | 64772610 | 38.74% | 52.91% | 87.53% | 95.54% |
| cg18315935 | TMEM53 | 1 | 44654623 | 44654624 | 98.23% | 99.01% | 94.10% | 98.85% |
| cg22274395 | UCP1 | 4 | 140568913 | 140568914 | 58.25% | 83.88% | 0.79% | 2.98% |
| cg27393285 | ZIM2-AS1 | 19 | 56765920 | 56765921 | 15.43% | 46.71% | 22.59% | 59.91% |
| cg27352056 | ZNF702P | 19 | 53013932 | 53013933 | 62.15% | 78.67% | 82.02% | 96.74% |
